# Weighted predictive modelling estimation of patient acceptable symptom state for forgotten joint score, Oxford hip score, and EuroQol health index 3 and 12 months after total hip arthroplasty in a United Kingdom cohort

**DOI:** 10.1007/s11136-026-04186-z

**Published:** 2026-02-12

**Authors:** Glory Uche Abugu, Nicholas Holloway, Philip Riches, Jon Clarke, Mario Ettore Giardini, Swati Chopra

**Affiliations:** 1 Department of Biomedical Engineering, Wolfson Centre, University of Strathclyde, 106 Rottenrow East, Glasgow, G4 0NW UK; 2 Department of Orthopaedics, Golden Jubilee University National Hospital, Agamemnon St, Clydebank, G81 4DY UK; 3 School of Science and Engineering, University of Dundee, Fulton Building, Dundee, DD1 4HN UK

**Keywords:** Patient-reported outcome measures, Joint-specific scores, General health-related quality of life, Patient acceptable symptom state, Hip replacement

## Abstract

**Purpose:**

Patient acceptable symptom state (PASS) enhances interpretation of patient-reported outcome measures (PROMs). However, very few studies have defined PASS values for widely used PROMs and are based on methods sensitive to distribution of PROM scores or weak correlation. This study utilises a new anchor-based method to estimate PASS thresholds for Forgotten Joint Score (FJS), Oxford Hip Score (OHS) and EuroQol health index (EQ-5D-5L) at 3 and 12 months after primary total hip arthroplasty (PTHA).

**Methods:**

This retrospective cohort study used data for PTHAs performed at a high-volume arthroplasty centre in Scotland between April 2021 and March 2023. PROMs were FJS, OHS and EQ-5D-5L. A new weighted predictive modelling method was used to define PASS values. Anchor questions used were surgery-specific satisfaction for the FJS and OHS, and EQ-Visual Analogue Scale for the EQ-5D-5L.

**Results:**

A total of 2793 PTHAs were performed, of which 65 to 73% had complete PROMs data. Respective median age and BMI were 69 years and 29.1 kg/m² and 57% were female. The Spearman correlations between anchors and PROMs ranged between 0.35 and 0.54. PASS thresholds (95% CI) at 3 and 12 months respectively were 31.5 (29.5–33.9) and 38.3 (36.5–42.4) for FJS, 31.5 (29.1–32.8) and 36.2 (35.1–36.8) for OHS, 0.814 (0.795–0.822) and 0.867 (0.845–0.875) for EQ-5D-5L.

**Conclusion:**

We report new PASS thresholds for FJS, OHS and EQ-5D-5L 3 and 12 months following PTHA. These thresholds reflect the symptom (health) state at which an average Scottish patient considers their outcome acceptable.

## Abstract (plain language summary)

Patient-reported outcome measures (PROMs) are increasingly being used to capture patients’ perception of the impact of total hip replacement (THR) on their health-related quality of life. However, interpreting these scores in a clinically meaningful way is still a challenge. Patient acceptable symptom state (PASS) is a crucial way to interpret PROMs data. It specifies a threshold or cut-off value in a PROM, such that patients whose PROM scores are above the threshold are considered to have an acceptable outcome. Despite its usefulness, PASS values at early to mid-follow up for commonly used PROMs such as Oxford Hip Score (OHS), Forgotten Joint Score (FJS) and EuroQol Health Index (EQ-5D-5L) are scarce with existing studies relying on methods that are biased to distribution of PROM data or correlation strength between anchor variable and PROM score. In this study, we determined PASS values for the OHS, FJS and EQ-5D-5L at 3 and 12 months after THR using a new weighted predictive modelling approach and data from over 2000 patients in Scotland. Our results indicate that 3 and 12 months after THR respectively, an average patient in Scotland requires scores of at least 31.5 and 38.3 for the FJS, 32 and 36 for the OHS, and 0.814 and 0.867 for the EQ-5D-5L to perceive their outcome as acceptable. PASS thresholds increased between 3 and 12 months reflecting ongoing recovery process. Male patients require higher scores to consider their outcome satisfactory.

## Introduction

Total hip arthroplasty (THA) is a highly effective, well-proven treatment for end-stage hip osteoarthritis. It significantly relieves pain, restores joint function and improves quality of life whilst also demonstrating considerable implant longevity of up to 25 years in nearly 60% of patients [1]. Based on these impressive results, demand for THA continues to rise, further driven by an aging population and changing demographics [2–4]. This increasing surgical volume necessitates efficient and cost-effective methods to monitor outcomes.

With increasing numbers of patients awaiting THA and subsequent growing pressures on healthcare resources, patient-reported outcome measures (PROMs) have emerged as a cost-effective and efficient method for monitoring surgical outcomes [5, 6]. PROMs not only allow assessment of large patient populations with minimal resource utilisation, but they also align with the increasing emphasis on patient-centred care and value-based healthcare delivery. While traditional outcome measures like revision rates or complications can identify significant outliers, they do not adequately capture whether a procedure was satisfactory from a patient’s perspective.

Several validated PROMs are used to evaluate outcomes after THA, including joint-specific measures such as the Forgotten Joint Score (FJS) and Oxford Hip Score (OHS), as well as general health measures like the EuroQol 5-Dimension (EQ-5D-5L) and the EuroQol visual analogue scale (EQ-VAS) questionnaires. While these scoring systems are well-validated, interpreting their numerical values in a clinical context remains challenging. When studies report statistically significant improvements in PROMs, these changes may not always translate into outcomes that patients consider meaningful [7, 8]. Standardised outcome thresholds can help interpret scores providing practical benchmarks for clinical practice [9, 10]. While various thresholds for interpreting PROMs exist, the patient acceptable symptom state (PASS) has particular relevance as it identifies the threshold where an average patient considers their state acceptable, providing a reference value to guide conversations about a patient’s health state following a treatment.

Methods for estimating PASS and other clinical cut-offs for THA can be broadly categorised into anchor-based and distribution-based methods. Anchor-based methods reference PROMs scores to a patient-rated variable (anchor) and are preferred by the COnsensus-based Standard for selection of health status Measurement INstruments (COSMIN) group [11] demonstrating higher accuracy and less bias than distribution-based methods. Existing PASS thresholds are mostly derived from the receiver operating characteristic (ROC) curve, and have been criticised for less precision (wider confidence intervals) and sensitivity to imbalanced anchor class distribution [12, 13]. The alternative predictive modelling (PM) method, though demonstrating higher precision than the ROC, may still be sensitive to imbalanced anchor class distribution [12–14]. Variants of the PM method [13, 15] have been proposed to overcome the bias of imbalanced anchor class and unreliability of anchor variable. They were derived under the assumption of normally distributed PROM scores which limits reliability of results with recent studies highlighting a risk of bias in skewed data [16, 17]. Furthermore, reported thresholds vary based on follow-up periods and have limited geographic representation given known international variations in outcomes following arthroplasty due to differences in patient populations, healthcare delivery, and surgical practices [8, 18]. To address these gaps, we aimed to establish PASS thresholds for the FJS, OHS and EQ-5D-5L at 3 and 12 months after THA using a new weighted predictive modelling method.

## Materials and methods

### Study setting and participants

The study utilised data on unilateral primary total hip arthroplasty (PTHA) performed between April 2021 and March 2023 at the Golden Jubilee National Hospital, a high volume elective Scottish arthroplasty centre. Data on the PTHAs was identified retrospectively within the Clinical Outcome Report Structure (CORS) project database held at centre. There were 2793 unilateral PTHAs performed within the time period, of which 2786 and 2769 respectively were considered eligible for data extraction at 3 and 12 months follow-up. The ineligible cases were for patients who died prior to their 3 and 12 months follow-up (6 and 23 patients respectively) as well as one patient who was under 18 years of age at operation. In addition, 18 complex PTHAs were performed during the same period. Complex PTHAs are defined as procedures in which revision type implants were deemed necessary due to extensive bone loss or gross instability in a primary setting. Given that these procedures represent an extreme and are atypical of routine primary procedure, they were excluded to avoid skewing the data.

### Data collection

The study centre routinely provides a set of PROM questionnaires to patients (via post or telephone) before their procedure and subsequently at 3 months, 1 year, 3 years and 5 years after surgery. The exception to this is the FJS which is only completed postoperatively in keeping with the original rationale for its development as an assessment tool for “awareness” of the ‘artificial joint’. Responses to the questionnaires are recorded within the CORS project database held at the centre. Data on PROMs, patient characteristics and operative factors were extracted from the database. The research team handled all data in accordance with the United Kingdom Caldicott principles while adhering to the Strengthening the Reporting of Observational Studies in Epidemiology (STROBE) guideline [19].

### Indications for THA, surgical technique and complications

All procedures at our high-volume teaching hospital were performed by 42 surgeons (17 consultants and 25 trainees). All our consultants are considered high-volume (> 30 cases per year). The majority of cases were performed by the consultant as first operator (69%), with a smaller proportion carried out by senior trainees under direct supervision. A standard posterior approach was used for 91% of the cases, the rest were done using an antero-lateral approach as per the routine practice of the surgeon. Fixation type varied in accordance with surgeon preference; hybrid (82%), cementless (13%), and cemented (5%). Early mobilisation was encouraged in line with an established departmental “Enhanced Recovery” protocol. If appropriate, some patients were placed on our “Day case” hip pathway, with the majority going home on post-operative day 1 or 2. Post-discharge, all patients were instructed on self-directed rehabilitation exercises and received a follow-up call from a trained arthroplasty practitioner at 10–14 days and 3 months to ensure there were no concerns. If necessary, patients were called back for face-to-face review or had the option of an “opt-in” review appointment at any stage if they had concerns. Indications for THA in majority of the cases was due to primary osteoarthritis (95%). Other indications were secondary osteoarthritis (1.1%), other inflammatory arthritis (1.3%), developmental dysplasia of the hip (0.9%) and avascular necrosis (0.8%). The overall recorded “complication” rate was 3% (71 out of 2035 cases). This included readmission (*n* = 18), dislocation/instability (*n* = 16), infection (*n* = 10), thromboembolic disease (*n* = 8), acute kidney injury (*n* = 6), revision (*n* = 5), wound complication (*n* = 4) periprosthetic fracture (*n* = 3) and vascular injury (*n* = 1).

###  Outcome measures

The outcome measures assessed in this study are the FJS, OHS and EQ-5D-5L health index. The FJS comprises 12 questions about artificial joint awareness during daily activities, responses are scored on a 0 to 4 scale, then transformed to a 0 to 100 (worst-best) scale [20]. OHS is composed of 12 questions about pain and function, scored from 0 to 4 and summed to a total of 0 to 48 (worst-best) [21]. EQ-5D-5L measures five health dimensions (mobility, selfcare, usual activities, pain/discomfort, anxiety/depression) with five response levels. The EQ-5D-5L health index was calculated using the UK value set, generating an index from − 0.285 to 1 (death = 0, perfect health = 1, negative values indicate a state worse than death) [22].

**Fig. 1:**
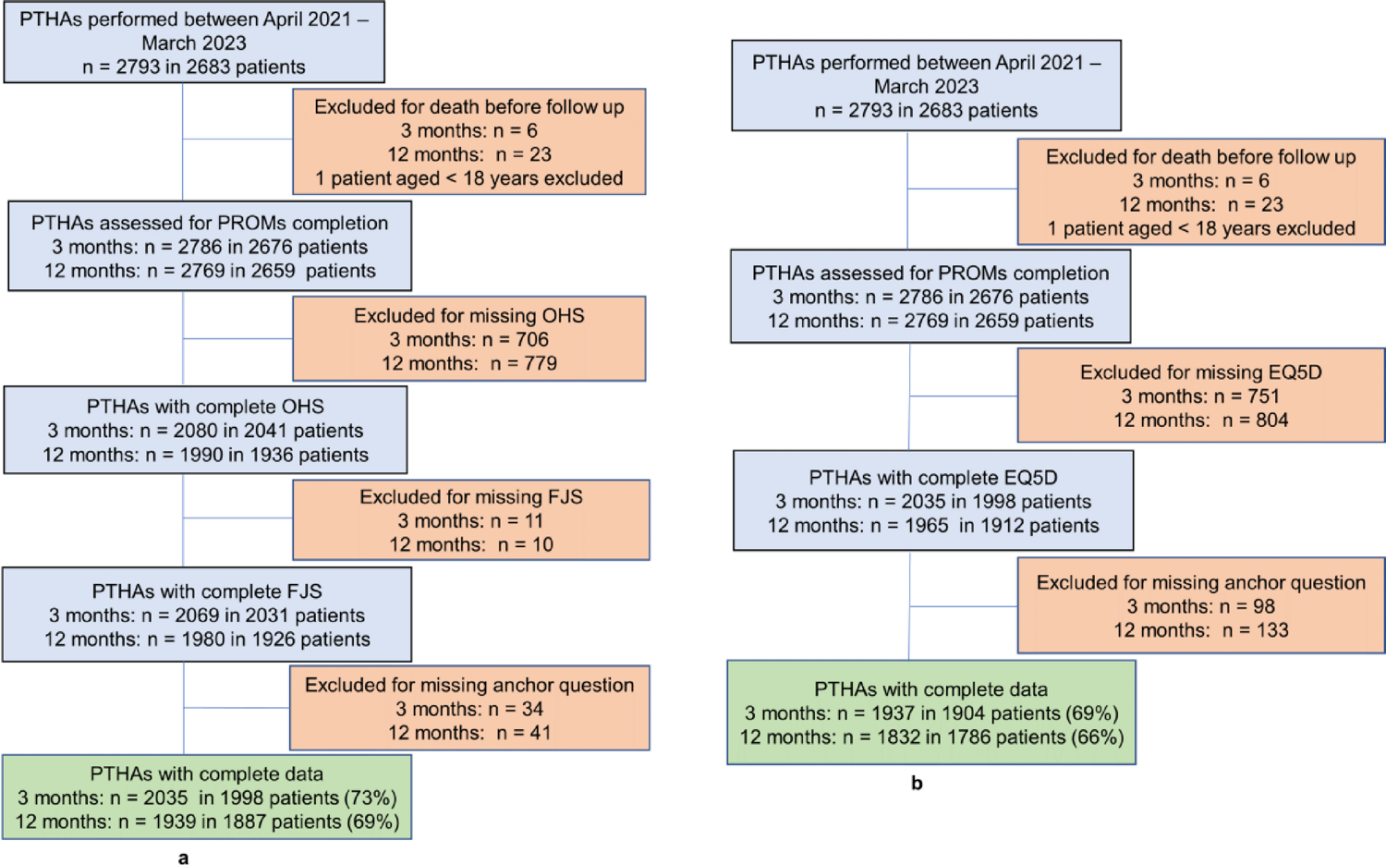
Flowchart of study participants. **a** Hip-specific outcome measures namely, Forgotten Joint Score, Oxford Hip Score and Patient Satisfaction (anchor variable). **b** General health outcome measures namely, EuroQol Health Index and Visual Analogue Scale (anchor variable)

### Anchor questions

An anchor question is an easily interpretable question which establishes a connection between a PROM score and the patients’ health condition [23]. It can be condition -specific or focus on general health but should correlate with the corresponding PROM. We used two anchors in this study, Patient satisfaction and EQ-VAS. The former is a single item questionnaire which measures patient satisfaction with their operated hip (“How satisfied are you with your operated hip?”) with responses recorded on a four-point scale namely, “very satisfied”, “satisfied”, “unsure” and “dissatisfied”. The EQ-VAS, also referred to as health score, is a visual health thermometer that assesses the current health state of patients and is measured on a 0 to 100 scale. A score of 0 represents the worst health the patient can imagine and 100 the best health. Patient satisfaction was chosen as the anchor for the two hip-specific PROMs (FJS and OHS) since it also assesses hip-specific outcome and is commonly used as anchor for estimating PASS for various joint-specific outcomes [23–26]. Conversely, the EQ-VAS was used as the anchor for the EQ-5D-5L health index to match the domain of general health-related quality of life which the EQ-5D-5L health index also assesses. Previous studies have found that the most suitable anchors focus on the same domain (generic or condition-specific) as the corresponding PROMs noting that hip-specific outcomes cannot be used as anchor to assess general health PROMs [23, 27]. Responses to anchor questions were dichotomised into acceptable symptom state and unacceptable symptom state. Patients responding “very satisfied” or “satisfied” to patient satisfaction question were classified as reporting a hip-specific acceptable symptom state (PASS group) while those answering “unsure” or “dissatisfied” were classified as reporting a hip-specific unacceptable symptom state (Non-PASS group) [24, 25]. If a patient scored 80 or above in the EQ-VAS, they were considered to have an acceptable general health state and classified as PASS group. This cut-off is consistent with the UK population health state classification [28], EQ-VAS PASS validation studies [23, 27], EQ-VAS mean of 79 and median (IQR) of 80 (70–90) observed in the study cohort which also align with those from large national registries such as UK NHS PROMs and the Swedish hip arthroplasty registry [29, 30].

### PASS threshold estimation

In order to estimate PASS values for the PROMs, the study utilised a new weighted predictive modelling (WPM) method recently developed as a simple and practical alternative to the predictive modelling (PM) method [12].

The WPM method [16] extends the PM method by applying a weighted likelihood function of the logistic regression using weights derived from the rank difference between PROM score and original anchor responses. The weighted approach assigns higher weights to data that exhibit strong positive correlation between the anchor and PROM score and lower weights to those exhibiting weak or negative correlation. The motivation for the weighted approach is to overcome the susceptibility of anchor-based methods to high misclassification rates (false positives and negatives) when correlation between the anchor and PROM score is weak (*r* < 0.4). Moreover, the WPM demonstrates high accuracy when the proportion of patients reporting satisfactory symptom state deviates from 0.5 under both normal and skewed distribution of PROM scores [16].

The PM and WPM methods were originally developed to assess threshold for meaningful within-individual change which describes the smallest meaningful change between pre- and post-operative outcome scores perceived by a patient after an intervention such as THA. The PM method is based on a logistic regression in which the dependent variable is a binary anchor variable and the independent variable is the difference between pre- and post-operative scores. PASS on the other hand defines a threshold in a post-operative outcome score above which a patient is considered to have experienced a satisfactory outcome following an intervention. The PM and WPM methods are easily adapted for PASS estimation by changing the independent variable in respectively logistic regression models from the change in outcome scores to the post-operative score at the follow-up time being considered. We used the original responses of the EQ-VAS with values ranging from 0 to 100 for specifying the weight in the WPM. However, for patient satisfaction, the dichotomised anchor responses were used to minimise any potential biases related to the lack of symmetry in the response scale.

The validity of the chosen anchors was assessed by calculating Spearman’s correlation between the dichotomised anchors and the PROM scores, and a correlation coefficient within the range of 0.3 to 0.7 was considered appropriate [31]. Non-parametric bootstrapping (*n* = 1000) was used to calculate 95% confidence intervals for the PASS estimates reported as 0.025 and 0.975 quantiles. Achievement rates of the estimated PASS thresholds calculated as percentage of patients whose scores were greater or equal to the thresholds were compared with the actual PASS percentage (percentage in the PASS group). Sensitivity and specificity were calculated using contingency tables. Empirical cumulative distribution functions (eCDFs) were plotted separately for the PASS and non-PASS groups while marking the PASS thresholds estimated in the study, as recommended by the U.S Food and Drug Administration [32]. To investigate whether PASS thresholds vary by patient characteristics, data was stratified by gender (male and female) and age (> 69 and ≤ 69 years) based on the median age of the study cohort, then PASS thresholds were estimated for each subgroup.

**Table 1:** Comparison of pre-operative demographics between patients with complete proms data and those with missing data. Values are median (interquartile range), unless otherwise stated

Hip-specific PROMs data	3 months data		12 months data		*p*-value^*^
	Completers (*n* = 2035)	Non-completers (*n* = 751)	Completers (*n* = 1939)	Non-completers (*n* = 830)	
Age	69 (62–75)	65 (57–72)	69 (62–75)	65 (57–73)	< 0.001
Female, n (%)	1165 (57)	441 (59)	1110 (57)	485 (58)	0.477^a^, 0.553^b^
BMI	29 (26–33)	30 (26–34)	29 (26–33)	30 (27–34)	< 0.001
General health PROMs data	Completers (*n* = 1937)	Non-completers (*n* = 849)	Completers (*n* = 1832)	Non-completers (*n* = 937)	
Age	69 (62–75)	65 (57–73)	69 (62–75)	66 (58–74)	< 0.001
Female, n (%)	1104 (57)	502 (59)	1047 (57)	548 (58)	0.288^a^, 0.492^b^
BMI	29 (26–33)	30 (26–34)	29 (26–33)	30 (26–34)	0.001^a^, 0.002^b^

*Wilcoxon Signed Rank test for continuous variables and chi-square test for categorical variables

^a^ Data at 3 months follow-up, ^b^ Data at 12 months follow-up

### Data analysis and presentation 

Patient characteristics were presented as median and interquartile range for continuous variables and frequencies and percentages for categorical variables. Normality was assessed using Shapiro-Wilk tests which found the data to show a non-normal distribution. Group differences were assessed using Wilcoxon Signed Rank test for continuous variables and chi-square test for categorical variables. All statistical analyses were performed using R version 4.4.3.

**Table 2:** Descriptive characteristics of outcome scores reported at 3 and 12 months follow-up

PROMs		3 months data			12 months data		
	Statistics	Entire cohort	PASS group	Non-PASS group	Entire cohort	PASS group	Non-PASS group
FJS	Count (%)	2035 (100)	1912 (94)	123 (6)	1939 (100)	1772 (91)	167 (9)
	skewness	−0.2	−0.2	1.9	−0.6	−0.7	1.5
	median	60.4	62.5	11.4	72.9	77.1	14.6
	IQR	33.3–81.8	35.4–83.3	2.2–25.0	39.6–93.8	49.5–95.8	2.2–27.1
	Mean (SD)	57.1 (29.1)	59.7 (27.7)	16.9 (18.7)	65.4 (31.1)	69.7 (28.2)	19.2 (20.3)
OHS	Count (%)	2035 (100)	1912 (94)	123 (6)	1939 (100)	1772 (91)	167 (9)
	skewness	−1.2	−1.3	−0.2	−1.6	−1.7	−0.2
	median	39	40	22	44	45	24
	IQR	32–44	34–45	16–29	36–47	39–48	16–31
	Mean (SD)	37.1 (9.4)	38.1 (8.6)	22.1 (8.6)	40.2 (9.4)	41.7 (7.7)	23.6 (10.1)
EQ-5D-5 L	Count (%)	1937 (100)	1241 (64)	696 (34)	1832 (100)	1143 (62)	689 (38)
	skewness	−2.1	−2.6	−1.4	−2.2	−3.1	−1.3
	median	0.866	0.922	0.751	0.922	1.0	0.753
	IQR	0.751–0.95	0.829–1.0	0.619–0.837	0.777–1.0	0.887–1.0	0.593–0.887
	Mean (SD)	0.817 (0.2)	0.888 (0.1)	0.692 (0.2)	0.841 (0.2)	0.929 (0.1)	0.695 (0.3)

FJS, forgotten joint score; OHS, oxford hip score; EQ-5D-5L, EuroQol five dimension five level; IQR, interquartile range; SD, standard deviation; PASS group, patients who reported acceptable symptom state based on hip-specific anchor (patient satisfaction) for FJS and OHS or acceptable general health state based on general health anchor (EQ-VAS) for the EQ-5D-5L

## Results

### Patient characteristics

A total of 2793 unilateral PTHAs was performed between April 2021 and March 2023, of which 2786 and 2769 were eligible for extraction for estimating PASS at 3 and 12 months follow-up respectively. PROMS data completion rates ranged from 66% to 73% (Fig. 1). Patient demographics were recorded during preoperative assessment. Patients with complete data had a median age of 69 years, median BMI of 29 kg/m² and 57% were female. These patients were 3–4 years older and had lower BMI (1 point) than those with incomplete data (Table 1). The percentage of patients reporting acceptable symptom state at 3 and 12 months respectively were 94% and 91% for joint-specific PROMs and 64% and 62% for general health PROM (Table 2).

**Table 3:** PASS thresholds with 95% confidence interval estimated using weighted predictive modelling with hip-specific patient satisfaction as anchor for FJS and OHS, and EQ-VAS as anchor for the EQ-5D-5L

	3 months data			12 months data		
	*r*	PASS	SNS/SPS	*r*	PASS	SNS/SPS
FJS	0.35	31.5 (29.5–33.9)	0.79/0.82	0.46	38.3 (36.5–42.4)	0.82/0.85
OHS	0.41	31.5 (29.1–32.8)	0.81/0.84	0.54	36.2 (35.1–36.8)	0.90/0.99
EQ-5D-5 L	0.47	0.814 (0.795–0.822)	0.80/0.68	0.52	0.867 (0.845–0.875)	0.80/0.72
FJS, forgotten joint score; OHS, oxford hip score; EQ-5D-5L, EuroQol five dimension five level health index; EQ-VAS, visual analogue scale of the EuroQol five dimension five level r, spearman rank correlation between dichotomised anchor and PROM, SNS, sensitivity; SPS, specificity						

### PASS thresholds

Moderate correlations were found between the PROMs and the dichotomised anchor with lowest correlation observed for FJS and data at 3 months follow-up (Table 3). PASS thresholds along with sensitivity and specificity are reported in Table 3. PASS achievement rates for the joint-specific PROMs were 76% for FJS and 77% and 75% for OHS at 3 months and 1 year respectively. For the EQ-5D-5L, 63% and 60% of patients as reached PASS at 3 and 12 months respectively.

**Fig. 2:**
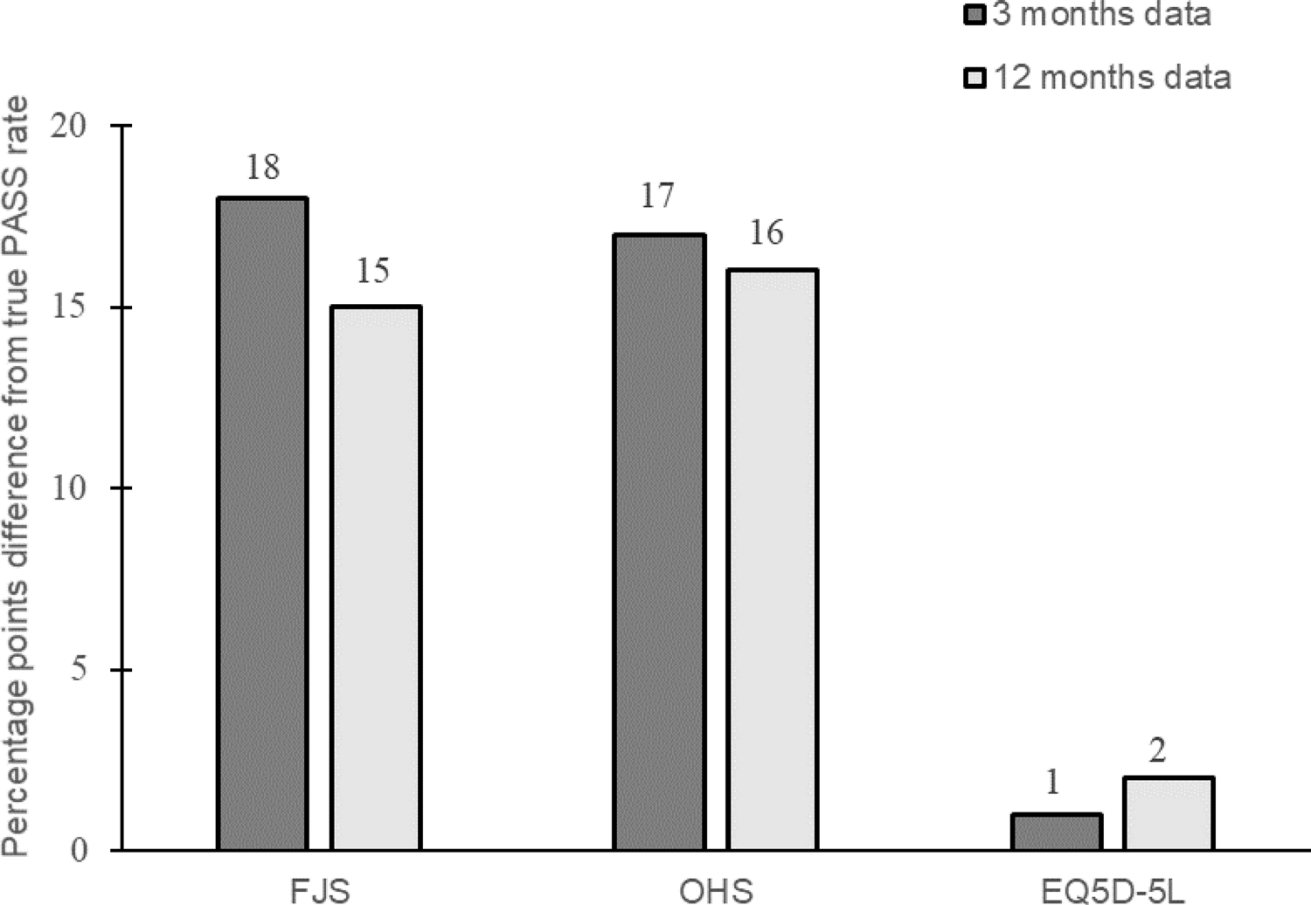
Difference in percentage points between true PASS percentage and percentage of patients achieving estimated PASS thresholds

The difference in percentage (percentage points [pp]) to the true PASS percentage was much larger for the joint-specific outcome scores compared to the generic EQ-5D-5L (Fig. 2). Precision as indicated by the 95% CIs tend to be lower in data where correlation between anchor and PROM score is weaker.

**Fig. 3:**
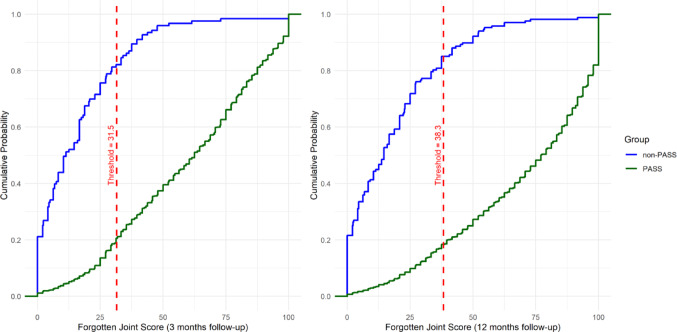
Empirical cumulative distribution functions (eCDF) for Forgotten Joint Score plotted separately for the PASS and non-PASS groups. Dashed red line indicates estimated PASS thresholds at x = 31.5 and x = 38.3 for 3 and 12 months follow-up timepoints respectively

**Fig. 4:**
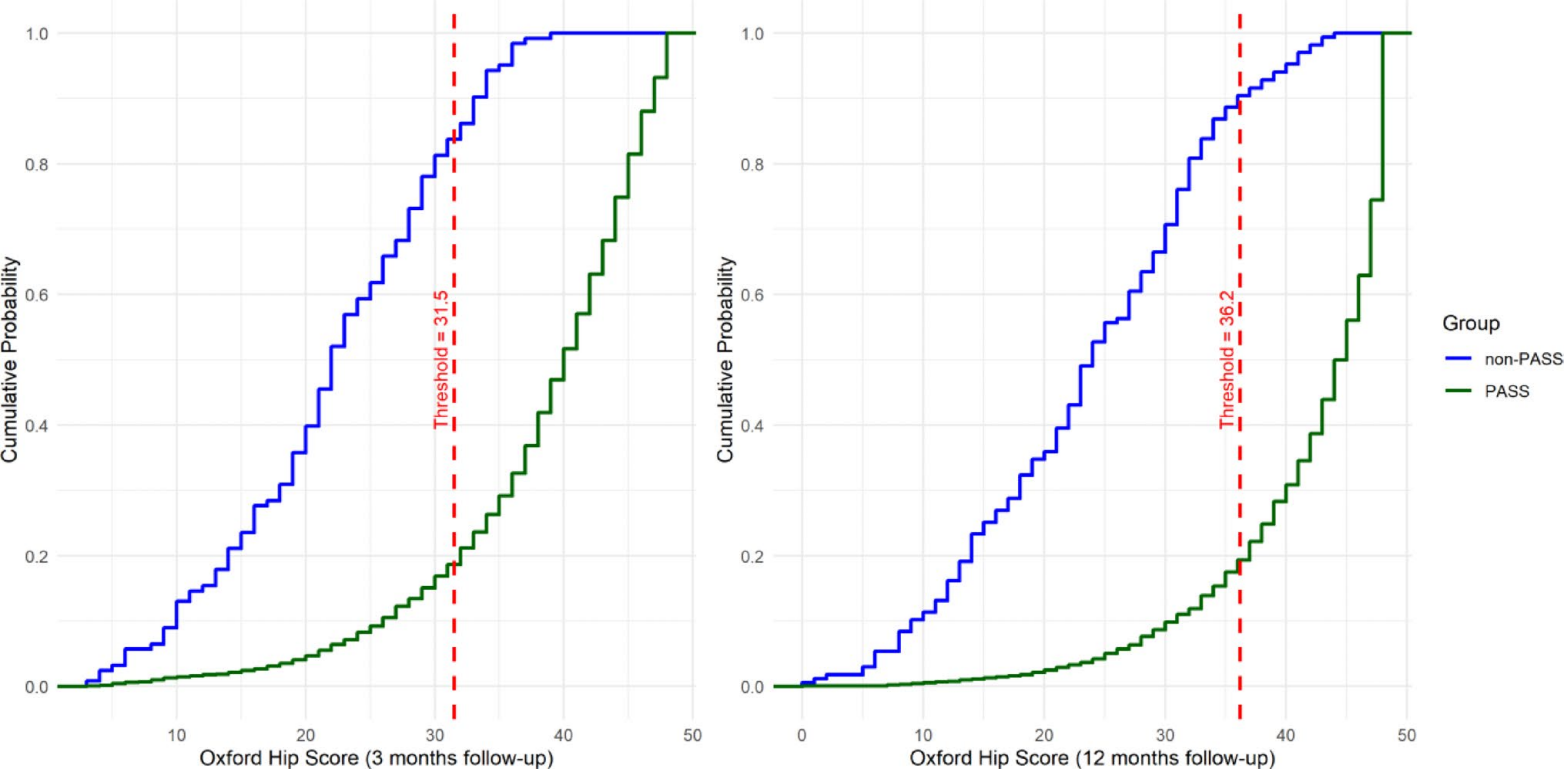
Empirical cumulative distribution functions (eCDF) for Oxford Hip Score plotted separately for the PASS and non-PASS groups. Dashed red line indicates estimated PASS thresholds at x = 31.5 and x = 36.2 for 3 and 12 months follow-up timepoints respectively

**Fig. 5:**
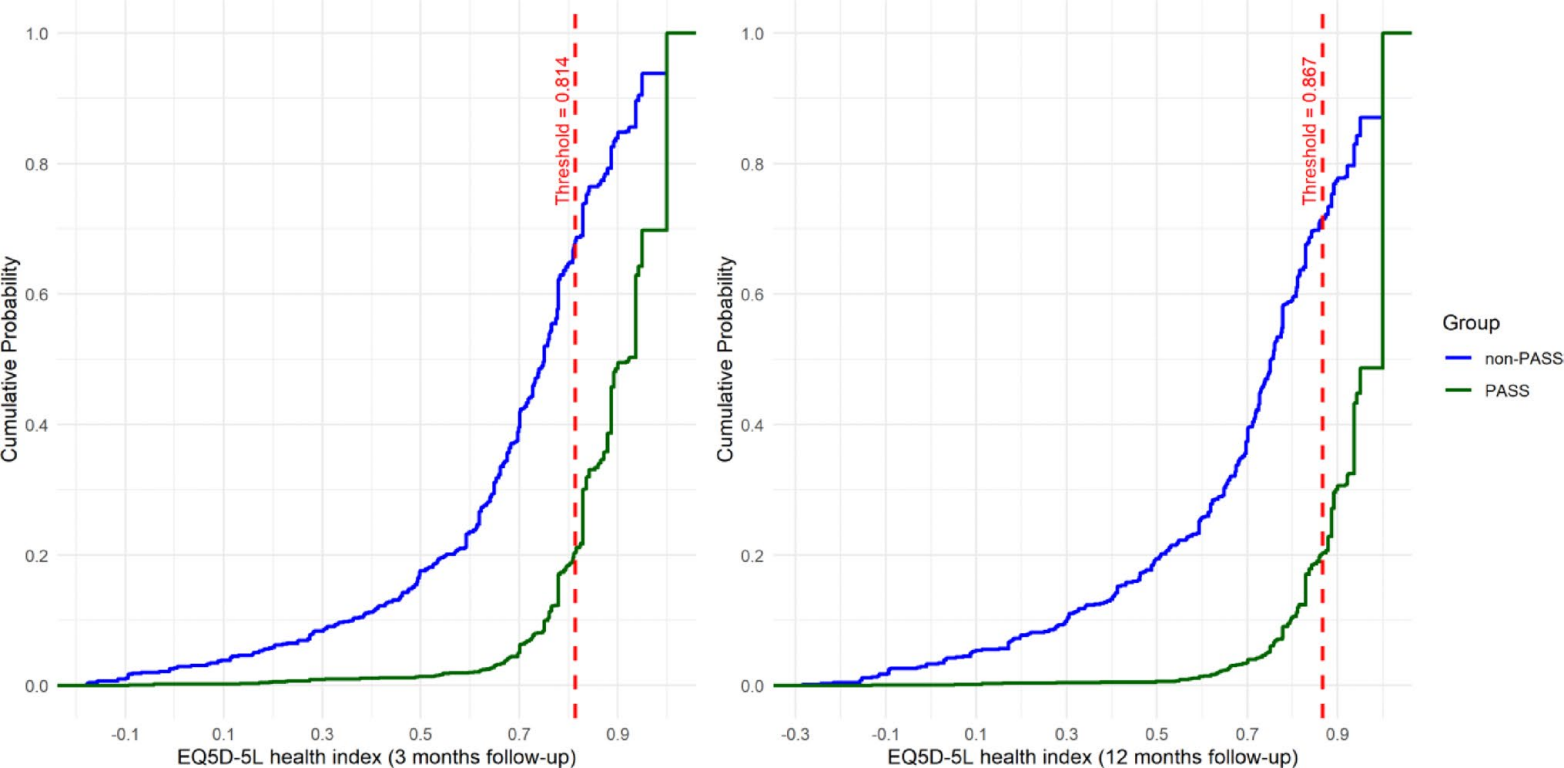
Empirical cumulative distribution functions (eCDF) for EQ-5D-5L health index plotted separately for the PASS and non-PASS groups. Dashed red line indicates estimated PASS thresholds at x = 0.814 and x = 0.867 for 3 and 12 months follow-up timepoints respectively

Estimated Cumulative distribution function (eCDF) plots stratified by PASS and non-PASS groups are presented in Figs. 3, 4 and 5. The proportion of patients in each group that achieved at least the estimated PASS thresholds can be read visually by tracing horizontally to the y-axis the point at which the eCDF plots intersect the vertical dashed line and subtracting the corresponding value from 1. For example, looking at Fig. 3, the proportions of patients in the PASS and non-PASS groups attaining the FJS PASS threshold are approximately 0.8 (1–0.2) and 0.2 (1–0.8) respectively.

**Table 4:** Subgroup PASS thresholds estimated using weighted predictive modelling with hip-specific patient satisfaction as anchor for FJS and OHS, and EQ-VAS as anchor for the EQ-5D-5L. Values in parentheses are 95% confidence interval

PROMs		3-months data		12-months data	
	Subgroup	*n*	PASS	*n*	PASS
FJS	Gender				
	Female	1165	31.3 (28.6–33.0)	1110	37.0 (35.1–41.7)
	Male	870	34.4 (30.3–37.2)	829	43.5 (37.6–47.0)
	Age				
	≤ 69 years	973	29.3 (25.7–31.5)	940	38.2 (33.6–42.0)
	> 69 years	1062	35.7 (32.6–37.8)	999	41.8 (37.8–44.7)
OHS	Gender				
	Female	1165	31.3 (28.7–32.0)	1110	34.1 (33.0–36.5)
	Male	870	31.5 (30.6–34.3)	829	36.4 (34.3–37.7)
	Age				
	≤ 69 years	973	31.9 (29.1–33.0)	940	36.6 (33.7–37.4)
	> 69 years	1062	30.8 (29.3–32.9)	999	35.3 (33.5–36.4)
EQ-5D-5 L	Gender				
	Female	1104	0.811 (0.795–0.825)	1047	0.865 (0.835–0.867)
	Male	833	0.815 (0.793–0.822)	785	0.867 (0.851–0.906)
	Age				
	≤ 69 years	926	0.811 (0.792–0.822)	896	0.865 (0.846–0.881)
	> 69 years	1011	0.817 (0.796–0.824)	936	0.858 (0.838–0.872)

FJS, forgotten joint score; OHS, oxford hip score; EQ-5D-5L, EuroQol five dimension five level; n, sample size.

### Subgroup analysis

PASS thresholds for age and gender subgroups are presented in Table 4. For gender, males exhibited higher thresholds in all three PROMs compared to females. Patients over 69 years had higher PASS thresholds in the FJS but lower thresholds in the OHS than those 69 years and under.

## Discussion

This study establishes new PASS thresholds for the FJS (31.5 to 38.3), OHS (31.5 to 36.2), and EQ-5D-5L (0.816 to 0.867) at 3 and 12 months following primary total hip arthroplasty using weighted predictive modelling approach in a large Scottish cohort of over 2,000 patients. Our findings demonstrate that PASS thresholds increased between early and mid follow-up periods for all three outcome measures, with joint-specific PROMs showing increases of 4.7 to 6.8 points and the EQ-5D-5L increasing by 0.053 points. Our findings showed that the percentage of patients that achieved the estimated PASS thresholds did not change for the FJS but decreased from 3 months to 1 year by 2 pp for the OHS and 3 pp for the Eq. 5D. Male patients had higher PASS thresholds than females. Patients over 69 years had higher FJS PASS thresholds but lower OHS PASS thresholds compared to those 69 years and under.

Surgery-specific satisfaction anchor used in estimating PASS for FJS and OHS classified 94% and 91% of patients as reporting acceptable symptom state with 75% to 77% of patients achieving the estimated PASS thresholds. In contrast, the general health anchor (EQ-VAS) used to estimate PASS for the EQ-5D-5L health index classified 64% and 62% of patients as having acceptable general health state while 63% and 60% reached the estimated PASS thresholds at 3 and 12 months respectively. Similar PASS achievement rates (74%) have been reported for the OHS in patient cohorts where satisfaction rates were equally high [33, 34]. Unlike direct satisfaction, the EQ-VAS captures broader aspects of patients’ quality of life that are unrelated to outcome of surgery, hence, the lower percentage of patients classified as having acceptable health state according to EQ-VAS. This aligns with the UK population norm where percentage of “healthy” patients was 56% [28]. Similarly, UK NHS PROMs data [29, 35] report 86–93% satisfaction rates and 93–97% pre-to-post surgery improvement in OHS and OKS, yet only 61–70% of patients report improvement in the EQ-VAS. This suggests that “high” rating in condition-specific outcomes do not necessarily translate to “high” perception in health state since other factors like comorbidities, contralateral joint problems and early recovery challenges can lower EQ-VAS scores even among “satisfied” patients.

The FJS PASS estimated in this study was substantially lower than previously reported thresholds. Earlier studies have documented PASS values of 66.7 and 92.2 points at 1 year in a US cohort, 69.8 to 76 points at 6 months in an Italian cohort, and 59 and 68 points at 3 and 12 months respectively in a Danish cohort [36–38]. These marked differences likely stem from methodological variations in choice of anchor questions (OHS, WOMAC and satisfaction) and estimation methods (ROC and 75th percentile). Our results align more with previously reported UK data, where a PASS threshold of 29 points was estimated at 6 months post-surgery [25] using the ROC method and a similarly-worded multi-category satisfaction anchor question. The results further suggest potential geographic variations in patient expectations and acceptable symptom state valuations in addition to methodological variations.

Our OHS PASS thresholds align closely with the 6-month PASS thresholds of 35.0 estimated based on a UK cohort using ROC method and a 0–100 VAS satisfaction anchor question cut-off of > = 50 [33]. Our PASS values were lower than previously reported at 1 year post-THA PASS from Denmark (*n* = 180, PASS = 40), Canada (*n* = 180, PASS = 39) and a German-speaking patient cohort from Germany and Switzerland (*n* = 193, PASS 41.5) using the ROC method [38–40] but higher than the adjusted predictive modelling PASS of 30.6 (*n* = 706) reported in a Danish cohort [41]. All the non-UK studies used a binary satisfaction/acceptability anchor question. The similarity of thresholds (difference of 1 to 2.5 points) obtained from the ROC-based studies despite differing in population coupled with disparity in thresholds (9.4 points) from the same Danish population using different estimation methods, highlight the strong influence of analytical approaches in the estimation of meaningful cut-off values.

The EQ-5D-5L PASS thresholds of 0.814 at 3 months and 0.867 at 1 year represent important reference values for the UK population and suggest markers of patient health state during post-surgery recovery. Reported PASS thresholds vary across studies: 0.87 to 0.92 for the EQ-5D-5L and 0.79 for the EQ-5D-3 L at 1 year in a Norwegian study [23, 27], 0.85 for the EQ-5D-5L at 1 year in a Canadian cohort [39], 0.76 for the EQ-5D-3L at 6 months and 1 year in a German-Swiss cohort [40] and 0.77 for the EQ-5D-3L in an international multi-centre study validated in a US institutional registry [42]. Anchor questions used include self-rated multi-category health state question [23, 27], binary satisfaction/acceptability question [39, 40], and numerical rating satisfaction question [42]. These variations reflect both cultural and healthcare system differences between countries, in addition to differences in the choice of anchor. Moreover, lower thresholds reported in studies using the EQ-5D-3L version [27, 40, 42] compared to those using the EQ-5D-5L in similar populations [23, 39] illustrate version-related effect, warranting caution when making international comparisons.

These thresholds have immediate clinical value, enabling surgeons to better interpret post-operative scores, guide conversation’s about patient expectations, and identify those who may warrant additional intervention. They also provide researchers with validated benchmarks for outcome studies and quality improvement initiatives. This large cohort study provides robust PASS thresholds for three commonly used PROMs following PTHA, making several important contributions to the field. We present the first early follow-up (3 months) PASS threshold for the OHS as well as the first UK-specific thresholds for the EQ-5D-5L and FJS at 3 and 12 months post-surgery, addressing a significant gap in the literature. In addition, our methodologically robust approach using weighted predictive modelling overcomes known limitations of traditional ROC and predictive modelling method, particularly in contexts with imbalanced anchor class and non-normally distributed PROMs data. Furthermore, our findings derive from a substantial cohort of over 2,000 patients, providing reliable benchmarks for clinical practice.

This study has a number of limitations. First, while our cohort size enhances statistical reliability, systematic differences between completers and non-completers in age and BMI may affect generalisability, with completers being older (69 vs. 65 to 66 years) and having lower BMI (29 vs. 30 kg/m^2^). Second, our single-centre design, while allowing for standardised data collection, may limit broader application although our patient characteristics align closely with those reported in the UK National Joint registry and Scottish Arthroplasty Project in terms of age distribution, gender proportion, and BMI ranges. Third, the study did not include pre-operative comorbidity data due to a change from paper-based system to an electronic system mid-way through the sample. Going forward as our sample size with electronic pre-operative comorbidity data increases, the ability to factor this in in future becomes possible. Fourth, the anchor (Patient Satisfaction) used for hip-specific PROMs had responses recorded on a four-point scale (“very satisfied”, “satisfied”, “unsure”, “dissatisfied”) which may introduce response bias due to the scale’s lack of symmetry. However, our dichotomisation strategy (grouping “very satisfied” and “satisfied” as PASS) maintains clinical relevance by focusing on patients who express clear satisfaction with their outcomes. Furthermore, the scale reflects the clinical questionnaire used in routine practice at our centre, meaning our findings provide thresholds applicable to the clinical tools actually available to practitioners in real-world settings. Fifth, while correlations between anchors and PROMs were moderate (*r* = 0.35 to 0.54), these values fall within the acceptable range for anchor-based methods [31], are consistent with those reported in similar orthopaedic PROM validation studies and reflect the complex, multifactorial nature of patient satisfaction in clinical practice. Sixth, the absence of pre-operative measurement limit comparative analysis of post-operative improvement. Lastly, although the weighted predictive modelling approach demonstrates better performance compared to the traditional PM, especially under a skewed PROM data distribution, it shows higher relative bias when correlation between the anchor and PROM score is weak [16] as is the case with the FJS at 3 months.

In conclusion, we have reported new PASS thresholds for three well-used PROMs using contemporary statistical method. Given the paucity of studies reporting PASS thresholds with methodological limitations in terms of sample sizes, follow- up timing, estimation method, and particularly sparse data from UK populations, our large cohort study provides robust thresholds for interpreting EQ-5D-5L, FJS, and OHS scores after THA. Thresholds for all 3 outcome scores increased between 3 and 12 months reflecting ongoing recovery process. Differences between our UK-derived thresholds and international values highlight how demographic and cultural factors influence acceptable symptom state thresholds emphasising the importance of population-specific benchmarks for clinical practice.

## Data Availability

The authors do not have permission to share data due to patient confidentiality.
